# Correlation between the Effects of Acupuncture at *Taichong* (LR3) and Functional Brain Areas: A Resting-State Functional Magnetic Resonance Imaging Study Using True versus Sham Acupuncture

**DOI:** 10.1155/2014/729091

**Published:** 2014-05-22

**Authors:** Chunxiao Wu, Shanshan Qu, Jiping Zhang, Junqi Chen, Shaoqun Zhang, Zhipeng Li, Jiarong Chen, Huailiang Ouyang, Yong Huang, Chunzhi Tang

**Affiliations:** ^1^School of Traditional Chinese Medicine, Southern Medical University, Guangzhou, Guangdong Province 510515, China; ^2^First Clinical School, Southern Medical University, Guangzhou, Guangdong Province 510515, China; ^3^Clinical Medical College of Acupuncture, Moxibustion and Rehabilitation, Guangzhou University of Chinese Medicine, Guangzhou, Guangdong Province 510405, China

## Abstract

Functional magnetic resonance imaging (fMRI) has been shown to detect the specificity of acupuncture points, as proved by numerous studies. In this study, resting-state fMRI was used to observe brain areas activated by acupuncture at the *Taichong* (LR3) acupoint. A total of 15 healthy subjects received brain resting-state fMRI before acupuncture and after sham and true acupuncture, respectively, at LR3. Image data processing was performed using Data Processing Assistant for Resting-State fMRI and REST software. The combination of amplitude of low-frequency fluctuation (ALFF) and regional homogeneity (ReHo) was used to analyze the changes in brain function during sham and true acupuncture. Acupuncture at LR3 can specifically activate or deactivate brain areas related to vision, movement, sensation, emotion, and analgesia. The specific alterations in the anterior cingulate gyrus, thalamus, and cerebellar posterior lobe have a crucial effect and provide a valuable reference. Sham acupuncture has a certain effect on psychological processes and does not affect brain areas related to function.

## 1. Introduction 

The traditional Chinese medical therapy, acupuncture, is used clinically as an alternative or supplementary treatment [1, 2]. A few studies have investigated the mechanism of action of acupuncture, with functional magnetic resonance imaging (fMRI) being an effective method to study the results of acupuncture [3–5]. The* Taichong* (LR3) acupoint has been used for the study of specificity of meridians and acupoints. Many studies have investigated the effects of acupuncture at LR3 on brain function using fMRI [6–9]. These studies were based on a block design using a general linear model. Because acupuncture has persistent effects [10], conclusions based on this model may show a false-positive phenomenon. In addition, the acupuncture mode described above is not fit for the clinic. To better simulate clinical acupuncture treatment, this study performed acupuncture at LR3 using resting-state fMRI and compared the preacupuncture and postacupuncture image data to verify the changes in brain functional connectivity in different brain areas in an attempt to explain the mechanism of action of acupoints.

Previous studies [6, 8, 9] concerning fMRI at LR3 have compared the difference in brain function between acupuncture at LR3 and surrounding areas (nonacupoint) and confirmed the specificity of meridians and points on brain function. However, some shortcomings were noted in these studies. The nonacupoint was near LR3, making it difficult to avoid blood vessels and nerve segments with similar regulatory effects. This activation of nerves or blood vessels could possibly affect study results in the specificity of meridians and points. In addition, the use of nonacupoints at different sites of acupuncture to verify the specificity of meridians and points has some limitations. A previous study [11] has demonstrated that different acupuncture methods at the same acupoint could induce various responses in the central nervous system. The acupuncture method used is important to demonstrate the specificity of acupuncture. This study used sham acupuncture as a control (blunt, nonpenetrating needles) and compared alterations in brain function after true acupuncture at LR3. The preacupuncture and postacupuncture brain areas affected were observed. This study compensated for the shortcomings of previous studies investigating LR3 by determining whether sham acupuncture exerted an identical effect to true acupuncture.

Previous studies mainly focused on cerebral functional imaging under acupuncture but seldom on effects after acupuncture [6–9]. This study focused on mechanisms of resting-state brain function as cerebral functional imaging studies have suggested that acupuncture can exert posteffects [10, 12]. Furthermore, the study of the posteffects of acupuncture excluded interference from sensing the needle body on cerebral functional imaging and is also more clinically relevant [13].

The combination of amplitude of low-frequency fluctuation (ALFF) and regional homogeneity (ReHo) was used in the present study. ReHo mainly reflects the synchronism of a time series in regional brain areas, but not signal intensity, and indirectly reflects the synchronism of spontaneous activity of local neurons in brain areas [14]. ALFF represents the intensity of blood-oxygen level-dependent signal in each voxel, directly reflecting the spontaneous activity of neurons [15]. Thus, this study investigated the specificity of LR3 by analyzing the common alterations in brain areas using ReHo and ALFF methods. On the basis of the theory described above, this study compared preacupuncture and postacupuncture at LR3 cerebral functional imaging and alteration-related brain areas. This study also compared cerebral functional imaging after true acupuncture and sham acupuncture at LR3 to test whether acupuncture at LR3 specifically affected brain areas by producing activation or deactivation. The effects of directly activated brain areas and their association with the mechanism of action of LR3 were then determined.

## 2. General Data 

A total of 15 healthy subjects were from universities and colleges in Guangzhou city, China. Inclusion criteria: (1) being between 21 and 28 years of age; right handedness; (2) having regular diet; minimal liquor, tobacco, tea, and coffee; normal sleeping patterns (before 12 a.m.); moderate size body mass index of 18.5–23.9 (Chinese); no history of nervous system disease; (3) having no pain (including dysmenorrhea) or insomnia within 1 month before the test; (4) having no metallic substances in the body, such as stents; (5) being fit for noise and hypothermia; no fear of confined spaces; (6) having not undergone acupuncture within 1 month before the test.

The present study contained nine males and six females, aged from 21 to 23 years (mean = 21.80 ± 0.56 years). Subjects weighed 46–72 kg (mean = 55.40 ± 8.35 kg) and were 160–180 cm (168.6 ± 6.81 cm) tall. All subjects gave full informed consent.

This study was approved by the Chinese Ethics Committee of Registering Clinical Trials (ChiECRCT-2012011) and registered in the Chinese Clinical Trial Register (ChiCTR-TRC-12002427).

## 3. Methods 

### 3.1. Trials and Processing Methods

The subjects first underwent sham acupuncture at LR3. Thirty minutes later, subjects were subjected to the second acupuncture (true acupuncture at LR3). These volunteers were not told the order of true or sham acupuncture. Subjects were asked to pass urine and stool prior to treatment. Volunteers' eyes were masked with eyeshades, and earplugs were simultaneously worn so their audiovisual system could not be stimulated.

### 3.2. Acupuncture Methods

Acupuncture was performed by the same experienced physician. Tubes (DONGBANG AcuPrime, Exeter, UK) and Huatuo needles 0.30∗(25 mm–45 mm) (Suzhou Medical Supplies Co., Suzhou, China) were used in this study.

1. Sham acupuncture at LR3: bilateral LR3 was localized according to Name and location of Acupoints: Chinese National Standards GB/T12346 [16]. Skin was sterilized with alcohol. In accordance with a previously published method of sham acupuncture [17], the auxiliary part of the tube was applied to the skin, with a sham needle placed in the tube over the acupoint. The sham needle was then tapped to make its tip touch the skin without puncturing it and was maintained in place for 30 min.
2. True acupuncture at LR3: bilateral LR3 were localized according to Name and location of Acupoints: Chinese National Standards GB/T12346 [16]. Skin was sterilized with alcohol. The tube needling technique was used: the needle handle was tapped softly using a forefinger. Puncturing depth was controlled. After removal of the tube, the needle was vertically punctured at 15 mm ± 2 mm. After developing needle sensation, twirling at an angle of 90–180° and frequency of 60–90 times/min and lifting and thrusting at a range of 0.3–0.5 cm and frequency of 60–90 times/min were conducted. After manipulating the needle for 1 min, the needle was held in place for 30 min. During the 30 min, physician repeated this manipulation for 1 min every 10 min.

### 3.3. fMRI Examination

Subjects were conscious, placed in a supine position, and asked to breathe calmly. The head was fixed with a foam mat, thus greatly reducing active and passive movement. Earplugs were used to reduce hearing and eyeshades were also used. After subjects rested for 15 min, the scan began.

Experiments were performed on a GE 3.0T MRI scanner with an 8-channel head coil. The MRI data (resting-state BOLD sequence) were collected at 15 min before needling and 15 min after withdrawing the needle. The scanning methods were identical between sham and true acupuncture.

1. Transverse T1-weighted image (T1WI) sequence: 1 min, 51 s, fast spin echo sequence; OAx T1 FLAIR, repetition time: 1750 ms/echo time: 24 ms, inversion time: 960 ms, field of view: 24 cm × 24 cm/Z, matrix: 320 × 224/number of excitations = 1, thickness: 5.0 mm/interval: 1.0 mm, 30 slices total, echo train length: 8, and bandwidth: 31.25.
2. Resting-state fMRI BOLD data collection: gradient echo-echo-planar imaging sequence scanning was conducted for 6 min in accordance with the following parameters: repetition time: 3000 ms/minimum, echo time: minimum, flip angle: 90, field of view: 240 mm × 240 mm, thickness: 5.0 mm/interval: 1.0 mm, 30 slices each time, and matrix: 96 × 96/number of excitations = 1.

### 3.4. Image Processing and Analytical Methods

Preprocessing was carried out using Data Processing Assistant for Resting-State fMRI (DPARSF; Chao-Gan and Yu-Feng, 2010, http://www.restfMRI.net), which is based on Statistical Parametric Mapping (SPM8; http://www.fil.ion.ucl.ac.uk/spm/) and Resting-State fMRI Data Analysis Toolkit (REST, Song et al., 2011. http://www.restfMRI.net) [18, 19]. This includes DICOM format conversion, removal of 10 time points before image scanning, time correction, correction of head movement, space standardization, and space smoothing. After preprocessing, 15 subjects were included in the statistical analysis.

 ReHo analysis: using REST1.8 software, linear tendency of the data after preprocessing (space standardization was completed, and space smoothing was not finished) was removed by linear regression. Time and curve were convolved using Hamming bandpass filtering. ALFF was extracted (0.01–0.08 Hz). Kendall's coefficient of concordance of each subject was computed, so each subject had a Kendall coefficient of concordance map, that is, ReHo map. This map was divided by the mean of the whole brain, and standardized ReHo was obtained and used in statistical analysis [18, 20, 21].
 ALFF analysis: using REST1.8 software, linear tendency of the data after preprocessing (space smoothing was completed) was removed by linear regression. Time and curve were convolved using Hamming bandpass filtering. ALFF was obtained (0.01–0.08 Hz). ALFF of each subject was computed, so ALFF maps were obtained. ALFF value was divided by the mean of the whole brain, and standardized ALFF was obtained [15].

### 3.5. Statistical Analysis

The data were analyzed with REST 1.8 software. Intragroup standardized ReHo and ALFF values were detected with paired* t*-test (AlphaSim correction *P* < 0.05; continuous voxel >85). Finally, the preacupuncture and postacupuncture differences between the alterations of ALFF and ReHo were obtained in subjects of the same group. Rest1.8 software Viewer was employed to identify the precise anatomical position in the brain with statistical significance on the corresponding MNI coordinate. The results were presented as images.

## 4. Results 

### 4.1. ALFF Analysis

**Figure 1:**
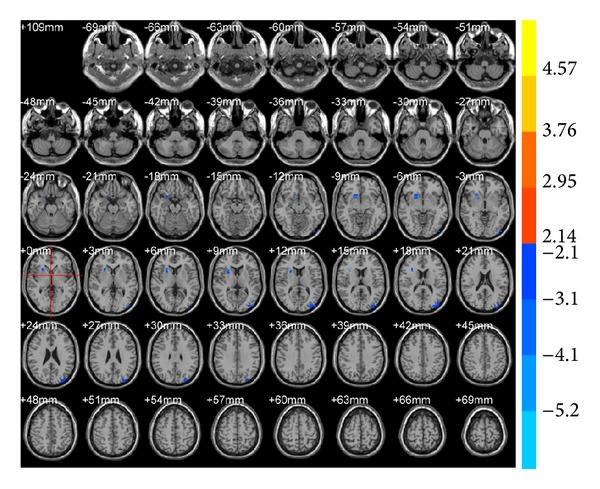
Brain areas with ALFF alteration after true acupuncture at LR3 versus preacupuncture. Blue represents deactivation; red represents activation; blank represents no activation.

**Figure 2:**
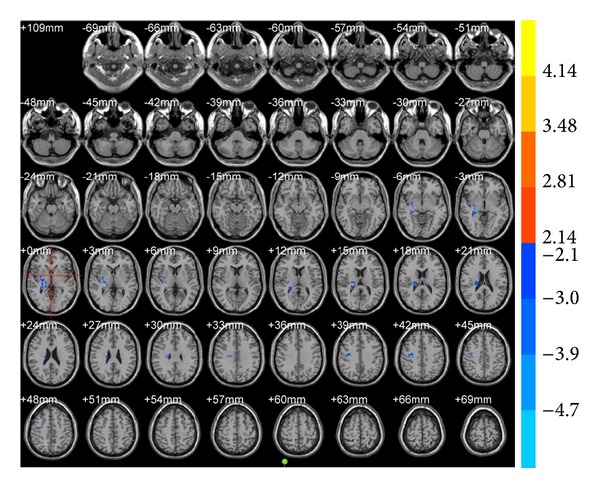
Brain areas with ALFF alteration after true acupuncture at LR3 versus sham acupuncture. Blue represents deactivation; red represents activation; blank represents no activation.

**Figure 3:**
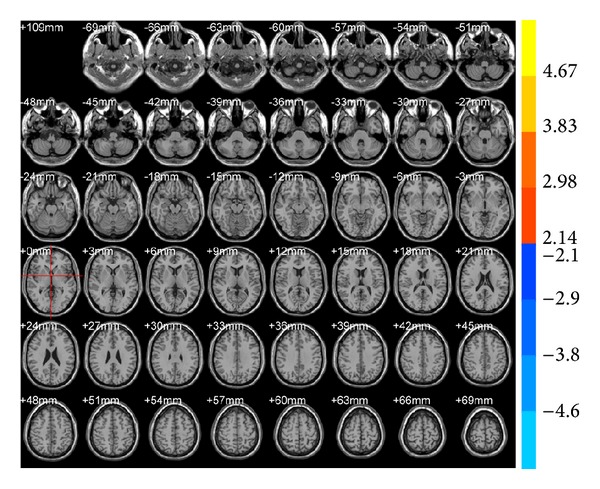
Brain areas with ALFF alteration after sham acupuncture at LR3 versus preacupuncture. Blue represents deactivation; red represents activation; blank represents no activation.

ALFF results showed that brain areas with alterations after true acupuncture at LR3 apparently decreased in the right frontal lobe (BA47) and left superior occipital gyrus (BA19;* T* value was negative; Table 1, Figure 1) versus preacupuncture. Brain areas decreased in the sublobar and extranuclear regions after true acupuncture compared with sham acupuncture at LR3 (Table 1, Figure 2). However, there were no functional areas with specific alterations after sham acupuncture at LR3 (Figure 3).

### 4.2. ReHo Analysis

**Figure 4:**
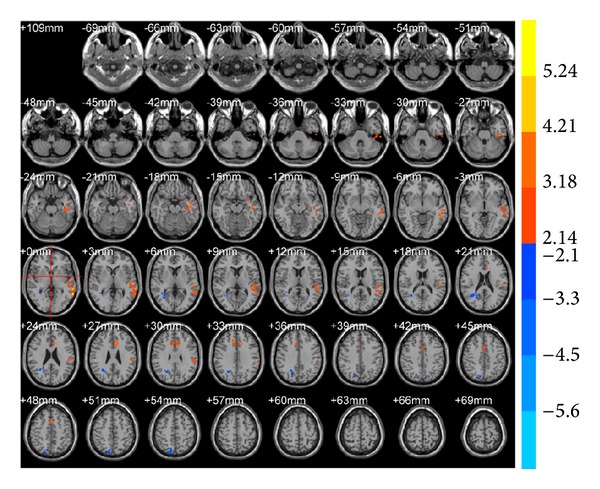
Brain areas with ReHo alterations after true acupuncture at LR3 versus preacupuncture. Blue represents deactivation; red represents activation; blank represents no activation.

**Figure 5:**
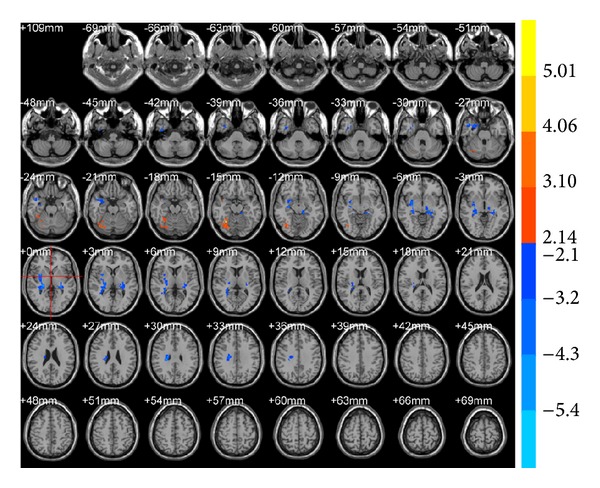
Brain areas with ReHo alterations after true acupuncture at LR3 versus sham acupuncture. Blue represents deactivation; red represents activation; blank represents no activation.

ReHo results demonstrated that, compared with preacupuncture, the right middle temporal gyrus (BA19) decreased (*T* value was negative) and the left inferior temporal gyrus (BA20), left middle temporal gyrus (BA21), and left anterior cingulate gyrus (BA32; Table 2, Figure 4) increased in brain areas after true acupuncture at LR3. After true acupuncture at LR3 ReHo showed that, in the right superior temporal gyrus (BA38), left cerebrum, sublobar and extranuclear regions, and right thalamus were decreased (*T* value was negative), while the right cerebellar posterior lobe was increased at LR3 compared with the sham acupuncture (Table 2, Figure 5).

**Figure 6:**
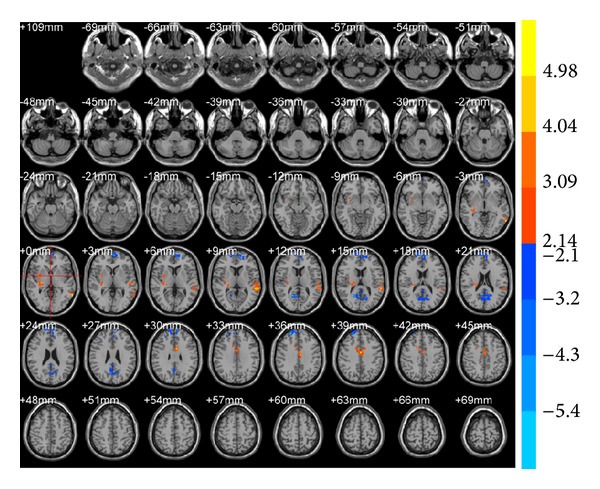
Brain areas with ReHo alterations after sham acupuncture at LR3 versus preacupuncture. Blue represents deactivation; red represents activation; blank represents no activation.

Comparing sham acupuncture at LR3 with preacupuncture, we found that decreased ReHo was visible in the right superior frontal gyrus (BA9) and right posterior cingulate (BA30). Enhanced ReHo was detectable in the left middle temporal gyrus (BA21), left cingulate gyrus (BA24), and right lenticular nucleus (Table 2, Figure 6). All these brain areas have been shown to be involved with association.

Both ALFF and ReHo results had signal deactivated in BA19 and sublobar and extranuclear regions.

## 5. Discussion 

After true acupuncture at LR3, brain areas with ALFF alterations included BA19 and BA47. Brain areas with ReHo alterations included BA19, BA20, BA21, and BA32. Deactivation in BA19 occurred with both ALFF and ReHo. BA19, a visual association area, can perceive and integrate visual information. Deactivation represents relative decrease in regional blood-oxygen signal intensity. A previous study verified that a decrease in blood-oxygen signal intensity may have been a mark of neuronal inhibition [22]. Another study also confirmed that the decrease in blood-oxygen signal intensity was associated with local field potentials and multiunit activity [23], primarily suggesting that acupuncture at LR3 specifically suppressed vision-related neurons. Siedentopf et al. [24] suggested that acupuncture at acupoints on the foot activated the visual cortex and treated vision-related disease. Simultaneously, an fMRI study confirmed that acupuncture at LR3 specifically activated BA19 [8]. Thus, we speculate that BA19 is a specific brain area for true acupuncture at LR3. Acupuncture at LR3 could bidirectionally regulate (excitation and inhibition) visual neurons, which could be a mechanism for treating vision-related diseases. Moreover, BA20 and BA21 activated by ReHo are associated with visual processing. This observation verified the bidirectional regulation on vision after puncturing LR3 and further confirmed that vision-related brain areas are specific for acupuncture at LR3. A previous study suggested that the anterior cingulate gyrus (BA32) participated in many complicated motor functions and pain reactions in the body [25]. Prior studies have also indicated that acupuncture at LR3 specifically activated the anterior cingulate gyrus [6, 8], indicating that the anterior cingulate gyrus was an additional specific brain area activated by acupuncture at LR3.

Brain areas after true acupuncture at LR3 versus sham acupuncture: ALFF alterations were visible in sublobar and extranuclear regions of the brain. ReHo alterations were observed in BA38, sublobar and extranuclear regions, thalamus, and cerebellar posterior lobe. ALFF and ReHo results demonstrated that deactivated brain areas included sublobar and extranuclear regions, but the functions of these brain areas were not prominent, and did not show close correlation with the effects of LR3 acupoint. This observation requires further investigation. The thalamus is a relay station buried under the cerebral cortex and is involved in sensory perception. A previous study showed that the thalamus was associated with the regulation of acute and chronic pain [26]. The thalamus is a component in the pain management network. This network was deactivated during development of needle sensation [11, 27], demonstrating that the thalamus could negatively regulate brain networks activated by pain. Thus, pain-activated brain areas were transformed into an inhibitory state, exerting an analgesic effect of acupuncture. Studies [6–9] have also found changes in the thalamus, indicating another area specifically altered after acupuncture at LR3. This observation provides further evidence for the analgesic efficacy of acupuncture at LR3. The main function of the cerebellar posterior lobe is to maintain balance, to regulate muscular tension, to coordinate voluntary movement, and to manage fine motor control. In clinical practice, acupuncture at LR3 could treat vertigo and lower limb paralysis, which could be associated with regulatory effects of LR3 on the vestibular nerve and spinal movement through the cerebellum. Moreover, the cerebellar posterior lobe is a component of the neocerebellum and has an extensive connection to the cerebral cortex. The neocerebellum is involved in affection and cognition [28], which could be a mechanism of LR3 acupuncture in the treatment of emotion-related disease. Numerous studies confirmed [29–31] that acupuncture activated the cerebellum, suggesting that this activation plays an important role in the mechanisms underlying acupuncture treatment.

ALFF results demonstrated that no brain areas were altered after sham acupuncture and reflected the specificity of meridians and acupoints from an indirect source. ReHo results revealed that, after sham acupuncture, alterations were observed in the superior frontal gyrus (BA9), posterior cingulate gyrus of the limbic lobe (BA30), cingulate gyrus of the limbic lobe (BA24), the left middle temporal gyrus (BA21), and the lenticular nucleus. Of these areas, the frontal lobe (BA9) mainly participates in prefrontal association; the middle temporal gyrus (BA21) participates in temporal association; the posterior cingulate gyrus (BA30) is mainly involved in limbic lobe association; and the cingulate gyrus (BA24) is mainly involved in emotion and cognition. These brain areas have been shown to be involved with association. It is presumed that sham acupuncture-induced sensation was different from that of a needle pricking skin. This difference would lead to thinking and association in most subjects, so association-related alterations were detected in these brain areas. The combination of ALFF and ReHo methods suggested that sham acupuncture at LR3 could not affect functional areas.

In this study, fMRI was performed 15 min after acupuncture. Activation and deactivation of brain areas further verified that acupuncture definitely caused posteffects. Moreover, true acupuncture at LR3 versus sham acupuncture specifically activated/deactivated relevant brain areas. This study primarily confirmed that regulatory effects and clinical efficacy of acupuncture were not the effects of psychological factors. The present study has some limitations. In this study, subjects were healthy. It is initially presumed that activated/deactivated brain areas are associated with the treatment of clinically relevant diseases. Whether it still has regulatory effects on brain areas in patients requires further investigation. Some brain areas activated/deactivated by LR3 acupuncture did not have clear functions. Moreover, these brain areas did not strongly associate with the effects of LR3, and we therefore did not deeply study these regions. Whether these areas exert specific effects of LR3 acupuncture also deserves further study.

In summary, using resting-state fMRI, true acupuncture at LR3 specifically activated/deactivated some brain areas related to vision, movement, sensation, emotion, and analgesia. These findings may show a mechanism underlying the regulatory effects of acupuncture at LR3. The results from the comparisons of posttrue acupuncture and pretrue acupuncture, as well as posttrue acupuncture and postsham acupuncture, confirmed that meridians and points (LR3) exert effects on specific brain areas, which were associated with the mechanism of action of LR3.

## Figures and Tables

**Table 1:** Brain areas with ALFF alteration.

Comparisons	Number of voxels	Brain areas	Right/Left	Brodmann area	Talairach (mm)			*T*
					*X*	*Y*	*Z*	
TA versus PA	101	Frontal lobe, subgyral	R	47	27	21	−3	−6.2209
	139	Superior occipital gyrus	L	19	−33	−87	30	−3.8911

TA versus SA	181	Sublobar, extranuclear	R		27	−36	15	−5.6675

**Table 2:** Brain areas with ReHo alterations.

Comparisons	Number of voxels	Brain areas	Right/Left	Brodmann area	Talairach (mm)			*T*
					*X*	*Y*	*Z*	
TA versus PA	134	Middle temporal gyrus	R	19	36	−57	18	−4.4085
	100	Temporal lobe, subgyral	L	20	−45	−21	−27	4.3696
	276	Middle temporal gyrus	L	21	−63	−51	0	6.1186
	118	Limbic lobe, anterior cingulate	L	32	−9	24	24	5.6813

TA versus SA	94	Superior temporal gyrus	R	38	36	9	−21	−4.0397
	115	Cerebellum posterior lobe, declive	R		33	−57	−15	5.21
	331	Sublobar, thalamus	R		24	−27	0	−6.5849
	129	Sublobar, extranuclear	L		−30	−24	−6	−4.8703

SA versus PA	101	Sublobar, lentiform nucleus	R		30	−18	0	4.5274
	267	Superior frontal gyrus	R	9	24	51	36	−6.5548
	184	Limbic lobe, posterior cingulate	R	30	18	−57	12	−5.514
	132	Middle temporal gyrus	L	21	−63	−54	0	5.3984
	109	Limbic lobe, cingulate gyrus	L	24	0	−6	39	5.4237
